# *Caulifigura coniformis* gen. nov., sp. nov., a novel member of the family *Planctomycetaceae* isolated from a red biofilm sampled in a hydrothermal area

**DOI:** 10.1007/s10482-020-01439-w

**Published:** 2020-06-24

**Authors:** Nicolai Kallscheuer, Sandra Wiegand, Christian Boedeker, Stijn H. Peeters, Mareike Jogler, Anja Heuer, Mike S. M. Jetten, Manfred Rohde, Christian Jogler

**Affiliations:** 1 Department of Microbiology, Radboud University, Nijmegen, The Netherlands; 2 Institute for Biological Interfaces 5, Karlsruhe Institute of Technology, Eggenstein-Leopoldshafen, Germany; 3 Leibniz Institute DSMZ, Brunswick, Germany; 4 Department of Microbial Interactions, Institute of Microbiology, Friedrich Schiller University, Jena, Germany; 5 Central Facility for Microscopy, Helmholtz Centre for Infection Research, Brunswick, Germany

**Keywords:** Marine bacteria, Mediterranean Sea, Biotic surfaces, *Planctomycetes*, Panarea

## Abstract

Pan44^T^, a novel strain belonging to the phylum *Planctomycetes*, was isolated from a red biofilm in a hydrothermal area close to the island Panarea in the Tyrrhenian Sea north of Sicily, Italy. The strain forms white colonies on solid medium and displays the following characteristics: cell division by budding, formation of rosettes, presence of matrix or fimbriae and long stalks. The cell surface has an interesting and characteristic texture made up of triangles and rectangles, which leads to a pine cone-like morphology of the strain. Strain Pan44^T^ is mesophilic (temperature optimum 26 °C), slightly alkaliphilic (pH optimum 8.0), aerobic and heterotrophic. The strain has a genome size of 6.76 Mb with a G + C content of 63.2%. Phylogenetically, the strain is a member of the family *Planctomycetaceae*, order *Planctomycetales*, class *Planctomycetia*. Our analysis supports delineation of strain Pan44^T^ from all known genera in this family, hence, we propose to assign it to a novel species within a novel genus, for which we propose the name *Caulifigura coniformis* gen. nov., sp. nov., represented by Pan44^T^ (DSM 29405^T^ = LMG 29788^T^) as the type strain.

## Introduction

*Planctomycetes* is a bacterial phylum displaying exceptional cell biological features (Rivas-Marin and Devos 2018; Wiegand et al. 2018, 2020). Together with *Chlamydiae*, *Verrucomicrobia* and other sister phyla, the phylum *Planctomycetes* forms the PVC superphylum and several of its members have environmental, medical or biotechnological relevance (Spring et al. 2016; Wagner and Horn 2006). The phylum itself is subdivided into the classes *Phycisphaerae*, *Planctomycetia* and *Candidatus* Brocadiae, which display differences in their cell biology, e.g. mode of cell division and metabolism (Wiegand et al. 2020). One example are species of *Cand.* Brocadiae, which perform unique reactions during anaerobic ammonium oxidation (anammox) (Strous et al. 1999). These reactions are e.g. exploited for converting ammonium to dinitrogen gas during *N*-elimination in wastewater treatment plants (Peeters and van Niftrik 2018). The class *Phycisphaerae* comprises strains that form spherical cells and divide by binary fission (Fukunaga et al. 2009). This is a decisive difference compared to budding as the observed mode of cell division in the other two classes. Similar to *Phycisphaerae*, species belonging to genera within the class *Planctomycetia* have been often isolated from aquatic biotic and abiotic surfaces (Bondoso et al. 2014, 2017; Kohn et al. 2016; Vollmers et al. 2017), on which they can be highly abundant (Bengtsson and Øvreås 2010). Such species likely use complex polysaccharides derived from biotic surfaces, in particular macroscopic phototrophs, as source of carbon and energy (Jeske et al. 2013; Lachnit et al. 2013). However, the dominance of planctomycetal species on such surfaces is remarkable given their rather slow growth compared to natural competitors in this ecological niche, e.g. members of the ‘*Roseobacter* group’ (Frank et al. 2014). Underlying mechanisms allowing Planctomycetes to compensate for lower growth rates may include the capability to produce bioactive small molecules (Graça et al. 2016; Jeske et al. 2016; Kallscheuer et al. 2019b), resistance against several antibiotics (Cayrou et al. 2010; Godinho et al. 2019) and a specialised machinery for the uptake and intracellular digestion of complex polysaccharides. The latter is suspected to be facilitated by unique pili-forming crateriform structures and an extremely enlarged periplasmic space (Boedeker et al. 2017).

The application of novel microscopic techniques and genetic tools for Planctomycetes (Jogler et al. 2011; Jogler and Jogler 2013; Rivas-Marin et al. 2016b) has given more detailed insights into their cell envelope architecture and the mode of cell division (Rivas-Marin et al. 2020a). Planctomycetes were shown to possess peptidoglycan (Jeske et al. 2015; van Teeseling et al. 2015), which led to the conclusion that the cell envelope architecture of Planctomycetes resembles that of Gram-negative bacteria (Boedeker et al. 2017; Devos 2014). Nevertheless, Planctomycetes are still exceptional. Members of the orders *Gemmatales*, *Isosphaerales*, *Planctomycetales* and *Pirellulales*, as well as of the class *Cand*. Brocadiae, divide by budding while binary fission is the observed mode of division in the class *Phycisphaerae* (Wiegand et al. 2020). All known Planctomycetes lack many of the canonical divisome proteins including the otherwise universal FtsZ (Jogler et al. 2012; Pilhofer et al. 2008, Rivas-Marin et al. 2016a). Given their fascinating cell biology, several novel strains have been described in the recent year (Boersma et al. 2019; Dedysh et al. 2019b; Kallscheuer et al. 2019a, 2019c, 2020a, 2020b, Kohn et al. 2020; Kovaleva et al. 2019; Kulichevskaya et al. 2019; Peeters et al. 2020; Rensink et al. 2020), which led to an updated taxonomy and more precise definition of threshold values of phylogenetic markers in the class *Planctomycetia* (Dedysh et al. 2019b; Kallscheuer et al. 2019c).

As an additional contribution, here we describe the novel strain Pan44^T^ isolated from a red biofilm sampled in the shallow-water hydrothermal vent system close to Panarea Island, Italy.

## Materials and methods

### Isolation of the novel strain and cultivation

M1 medium with 4-(2-hydroxyethyl)-1-piperazineethanesulfonic acid (HEPES) as buffering agent and additionally supplemented with *N*-acetyl glucosamine (NAG) and artificial seawater (ASW) was used for the isolation and cultivation of strain Pan44^T^. The medium, designated M1H NAG ASW, was prepared as described (Boersma et al. 2019). Isolation of strain Pan44^T^ from a red biofilm sampled in a hydrothermal area close to Panarea island (exact location 38.5568 N 15.1097 E) was previously described (Wiegand et al. 2020). The biofilm was isolated on the 9^th^ of September 2013. Briefly, a piece of the natural biofilm was scraped off into sterile natural seawater using single-use scalpels. 20 µL of the biofilm suspension was streaked on M1H NAG ASW agar plates containing 500 mg/L streptomycin, 200 mg/L ampicillin and 20 mg/L cycloheximide, which were incubated at 20 °C for at least four weeks. The 16S rRNA gene of colonies obtained was amplified by PCR and sequenced following an established protocol (Rast et al. 2017). This step was performed in order to ensure that isolated strains selected for further characterisation indeed represent members of the phylum *Planctomycetes*.

### Determination of pH and temperature optimum

Cultivations for determination of the pH optimum were performed in M1H NAG ASW medium with 100 mM HEPES for cultivations at pH 7.0, 7.5 and 8.0. For cultivation at pH 5.0 and 6.0 HEPES was replaced by 100 mM 2-(*N*-morpholino)ethanesulfonic acid (MES), whereas 100 mM *N*-cyclohexyl-2-aminoethanesulfonic acid (CHES) served as a buffering agent at pH 9.0 and 10.0. Cultivations for determination of the pH optimum were performed at 28 °C. Cultivations for determination of the temperature optimum were performed in standard M1H NAG ASW medium at pH 8.0. Cell densities were measured as optical density at 600 nm (OD_600_).

### Microscopy protocols

Phase contrast and field emission scanning electron microscopy (SEM) were performed as previously described (Boersma et al. 2019). Transmission electron microscopy was performed according to a previously published protocol (Kohn et al. 2016).

### Genome information

The genome and 16S rRNA gene sequence of strain Pan44^T^ are available from GenBank under accession numbers CP036271 and MK554532, respectively. Sequencing of the genome is described in a previous study (Wiegand et al. 2020).

### Phylogenetic analysis

16S rRNA gene sequence-based phylogeny was computed for strain Pan44^T^, the type strains of all described planctomycetal species (assessed in January 2020) including all isolates recently published and described (Boersma et al. 2019; Dedysh et al. 2019a, 2019b; Kallscheuer et al. 2019a, 2019c, 2020a, 2020b; Kohn et al. 2020; Peeters et al. 2020; Rensink et al. 2020). The 16S rRNA gene sequences were aligned with SINA (Pruesse et al. 2012) and the phylogenetic inference was calculated with RAxML (Stamatakis 2014) with a maximum likelihood approach with 1000 bootstraps, nucleotide substitution model GTR, gamma distributed rate variation and estimation of proportion of invariable sites (GTRGAMMAI option). Three 16S rRNA genes of bacterial strains from the PVC superphylum, outside of the phylum *Planctomycetes* (*Opitutus terrae*, acc. no. AJ229235; *Kiritimatiella glycovorans,* acc. no. NR_146840 and *Lentisphaera araneosa*, acc. no. NR_027571), were used as outgroup. For the multi-locus sequence analysis (MLSA), the unique single-copy core genome of the analysed genomes was determined with proteinortho5 (Lechner et al. 2011) with the ‘selfblast’ option enabled. The protein sequences of the resulting orthologous groups were aligned using MUSCLE v.3.8.31 (Edgar 2004). After clipping, partially aligned *C*- and *N*-terminal regions and poorly aligned internal regions were filtered using Gblocks (Castresana 2000). The final alignment was concatenated and clustered using the maximum likelihood method implemented by RaxML (Stamatakis 2014) with the ‘rapid bootstrap’ method and 500 bootstrap replicates. Five planctomycetal genomes from the order *Pirellulales* served as outgroup. The average nucleotide identity (ANI) was calculated using OrthoANI (Lee et al. 2016). The average amino acid identity (AAI) was calculated using the aai.rb script of the enveomics collection (Rodriguez-R and Konstantinidis 2016) and the percentage of conserved proteins (POCP) was calculated as described (Qin et al. 2014). The *rpoB* nucleotide sequences were taken from publicly available planctomycetal genome annotations and the sequence identities were determined as described (Bondoso et al. 2013). Upon extracting only those parts of the sequence that would have been sequenced with the described primer set, the alignment and matrix calculation was done with Clustal Omega (Sievers et al. 2011).

## Results and discussion

### Phylogenetic inference

**Fig. 1:**
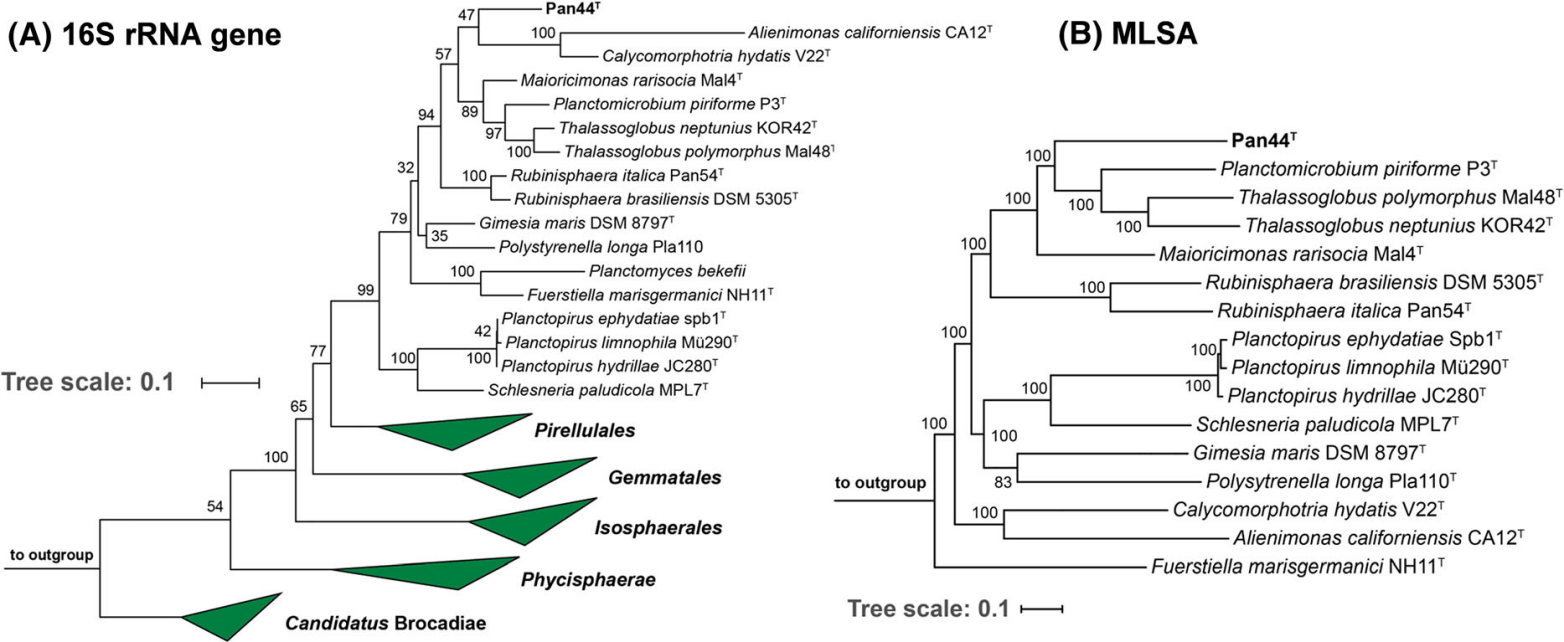
Maximum likelihood phylogenetic analysis. Phylogenetic trees showing the position of strain Pan44^T^. 16S rRNA gene- and MLSA-based phylogeny was computed as described in the Materials and methods section. Bootstrap values after 1000 re-samplings (16S rRNA gene)/500 re-samplings (MLSA) are given at the nodes (in %). The outgroup for the 16S rRNA-based tree consists of three 16S rRNA genes from the PVC superphylum. For the MLSA tree *Rubripirellula obstinata*, *Rhodopirellula baltica*, *Roseimaritima ulvae*, *Bythopirellula goksoyri* and *Thermogutta terrifontis* were used as outgroup

**Fig. 2:**
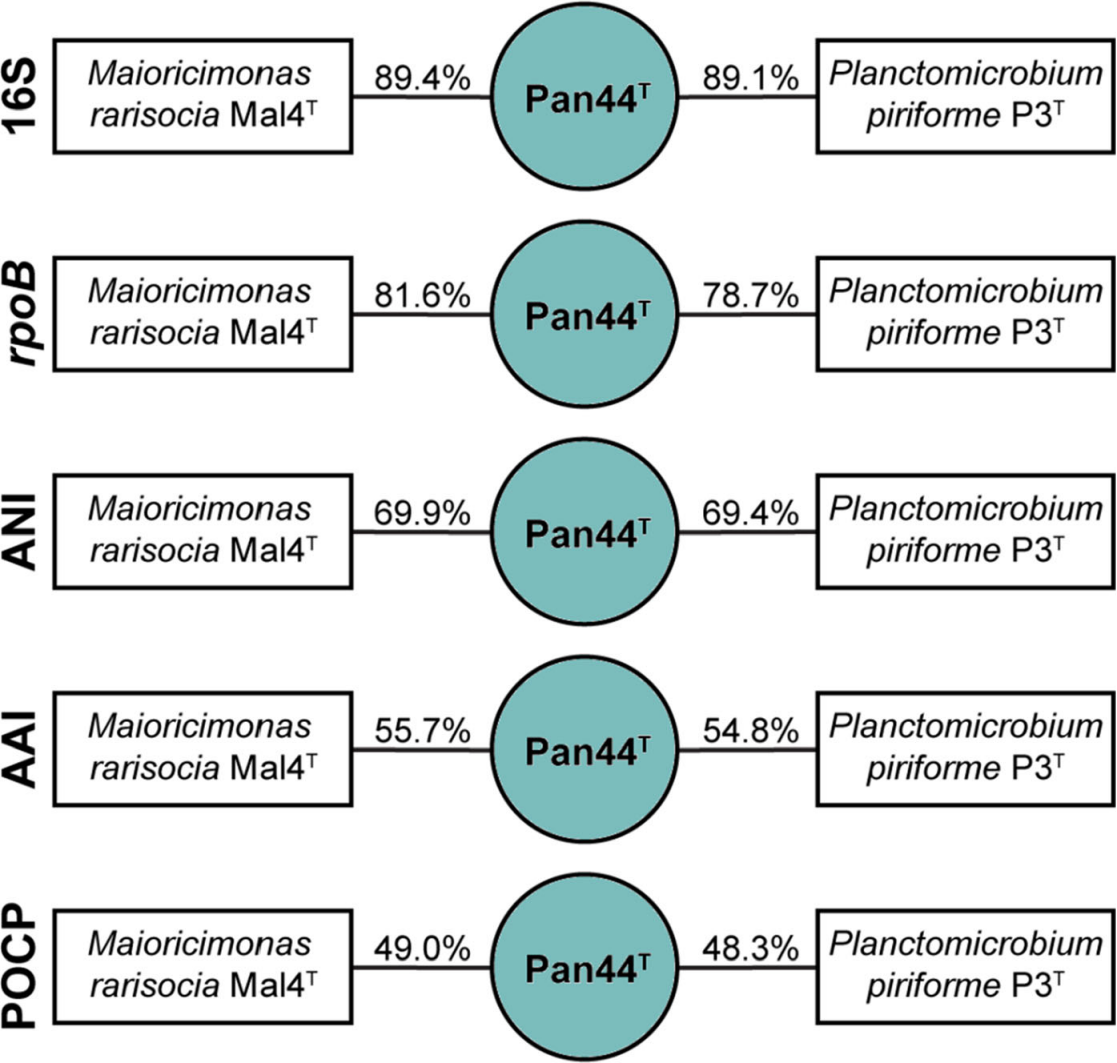
Analysis of phylogenetic markers. The figure shows the comparison of strain Pan44^T^ to its current closest relatives. Analysed markers include 16S rRNA gene identity (16S), *rpoB* gene identity, average nucleotide identity (ANI), average amino acid identity (AAI) and percentage of conserved proteins (POCP)

In the phylogenetic trees obtained from 16S rRNA gene sequence analysis and MLSA (Fig. 1), strain Pan44^T^ was observed to cluster within the family *Planctomycetaceae*, which is currently the sole family within the order *Planctomycetales*. All investigated phylogenetic markers (16S rRNA gene identity, *rpoB* identity, AAI, ANI and POCP) suggest *Maioricimonas rarisocia* Mal4^T^ and *Planctomicrobium piriforme* P3^T^ to be the current closest neighbours (Kulichevskaya et al. 2015; Rivas-Marin et al. 2020b). ANI values of 69.9% and 69.4%, respectively, indicate that strain Pan44^T^ is not a member of the species *M. rarisocia* or *P. piriforme*. The 16S rRNA gene sequence identity of strain Pan44^T^ compared to both strains is < 90% and thus falls below the proposed genus threshold of 94.5% (Yarza et al. 2014) (Fig. 2), thereby suggesting clear delineation of strain Pan44^T^ from members of the two genera. This conclusion is further supported by analysis of additional phylogenetic markers. Comparison of strain Pan44^T^ with *M. rarisocia* Mal4^T^ and *P. piriforme* P3^T^ yielded AAI and POCP values below the respective genus thresholds of 60% and 50%, respectively (Konstantinidis and Tiedje 2005; Qin et al. 2014) (Fig. 2). During analysis of a partial sequence of the *rpoB* gene (Fig. 2), we obtained identity values slightly above the proposed genus threshold of 75.5–78% (Kallscheuer et al. 2019c). This, however, should not overrule the overall conclusion based on the other phylogenetic markers, which are in line with the delineation of strain Pan44^T^ from known genera in the family *Planctomycetaceae*.

### Morphological and physiological analyses

**Fig. 3:**
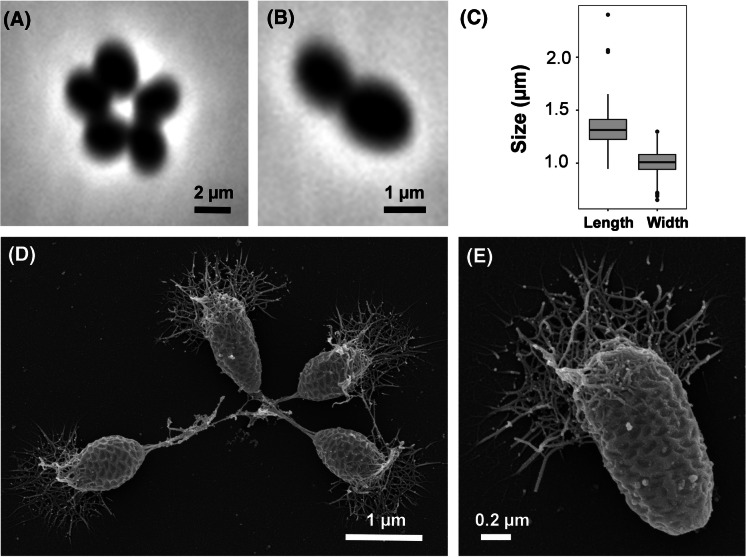
Microscopy images and cell size plot of strain Pan44^T^. The mode of cell division (**b**) and a general overview of cell morphology (**a**, **d**, **e**) is shown in the pictures, respectively. For determination of the cell size (**c**) at least 100 representative cells were counted manually or by using a semi-automated object count tool

**Fig. 4:**
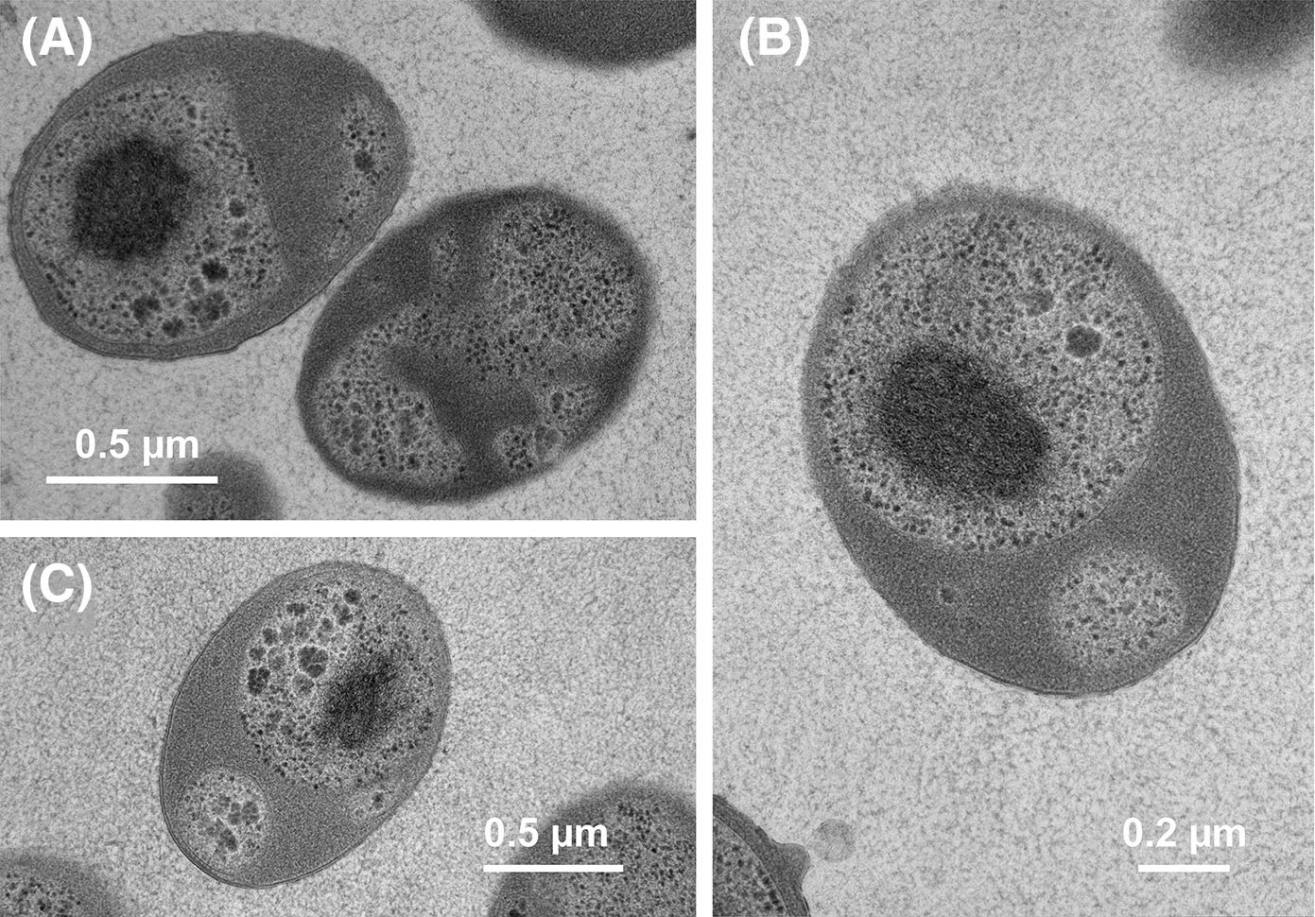
Thin sections of Pan44^T^ cells. Transmission electron micrographs show a condensed nuceloid and invaginations of the cytoplasmic membrane of Pan44^T^ cells. Separate scales bars are provided for each of the photographs

Basic features of strain Pan44^T^ regarding its physiology and morphology are summarised in Table 1 and compared to the close relatives *M. rarisocia* Mal4^T^ and *P. piriforme* P3^T^. For the analysis of morphological features, Pan44^T^ cells were harvested in the exponential growth phase and were analysed using phase contrast light microscopy and SEM (Fig. 3). Strain Pan44^T^ forms pear-shaped cells with an average size of 1.3 ± 0.2 × 1.0 ± 0.1 µm (Fig. 3a, c), which either appear as single cells or form rosettes or larger aggregates (Fig. 3d, e). Similar to *P. piriforme*, strain Pan44^T^ forms long stalks on one of the cell poles, which can reach a length of up to 0.8 µm (Fig. 3d). Stalks are particularly visible in smaller aggregates with less than ten connected cells. In contrast, stalks of *M. rarisocia* Mal4^T^ are much shorter. On the opposite pole of Pan44^T^ cells, fimbriae or matrix are usually formed. The cell surface has a characteristic texture comprised of triangles or rectangles, which resembles a pine cone (Fig. 3d, e). Such depressions can be artefacts of critical point drying during SEM specimen preparation. However, we optimised our preparation protocol for Planctomycetes and have never observed such an unusual pine cone texture in any other planctomycetal species described thus far (Boersma et al. 2019; Kallscheuer et al. 2019a, 2019c, 2020a, 2020b; Kohn et al. 2020; Peeters et al. 2020; Rensink et al. 2020). If SEM artefacts occur, planctomycetal cells tend to appear crescent-shaped, indicating that the pine cone texture of strain Pan44^T^ might be real rather than artefactual. Transmission electron micrographs of thin sections show typical planctomycetal features of Pan44^T^ cells, such as a condensed nucleoid and invaginations of the cytoplasmic membrane (Fig. 4).

**Table 1:** Phenotypic and genotypic features of strain Pan44^T^ compared to the closely related strains *Maioricimonas rarisocia* Mal4^T^ and *Planctomicrobium piriforme* P3^T^

Feature	Pan44^T^	*Maioricimonas rarisocia* Mal4^T^	*Planctomicrobium piriforme* P3^T^
Phenotypic features			
Shape	Pear-shaped	Pear-shaped	Ellipsoid to pear-shaped
Length (µm)	1.3 ± 0.2	2.0	1.7–2.8
Width (µm)	1.0 ± 0.1	1.4	0.9–1.3
Colour	White	Orange	White
Temperature range (optimum) (°C)	15–30 (26)	10–39 (31)	10–30 (20–28)
pH range (optimum)	5.0–10.0 (8.0)	6.5–9.0 (7.5)	4.2–7.1 (6.0–6.5)
Aggregates	Yes, rosettes	Yes, rarely	Yes, rosettes
Division	Budding	Budding	Budding
Dimorphic life cycle	n.o.	n.o.	Yes
Flagella	n.o.	n.o.	Yes
Crateriform structures	n.o.	Yes, overall	At reproductive pole
Fimbriae	Yes	Yes, overall matrix or fibre	Yes
Stalk	Yes	Yes	Yes
Holdfast structure	n.o.	n.o.	n.o.
Genomic features			
Genome size (bp)	6,761,146	7,744,989	6,317,004
Plasmids	No	No	n.o.
G + C content (%)	63.2	63.4	58.8 ± 1.7
Completeness (%)	96.55	98.28	95.69
Contamination (%)	1.72	0	1.72
Total genes	5587	5915	5117
Genes/Mb	826	764	810
Giant genes	0	1	1
Protein-coding genes	5524	5829	5050
Proteins-coding genes/Mb	817	753	799
Hypothetical proteins	2357	2257	2814
Coding density (%)	86.9	85.9	85.8
tRNAs	51	55	53
16S rRNA genes	2	2	1

The genome analysis is based on GenBank accession numbers CP036271, CP036275 and GCA_900113665.1, respectively. n.o. not observed

Strain Pan44^T^ divides by budding with the daughter cell having the same shape as the mother cell (Fig. 3b). Rosettes formed by strain Pan44^T^ look similar to those formed by *P. piriforme*, while *M. rarisocia* Mal4^T^ mostly occurs in the form of single cells and only in rare cases forms aggregates. Cell width for the three compared strains is similar, however, cells of strain Pan44^T^ are slightly shorter. Strain Pan44^T^ and *P. piriforme* P3^T^ lack pigmentation, whereas *M. rarisocia* Mal4^T^ is one of the rare examples of an orange-pigmented Planctomycete.

**Fig. 5:**
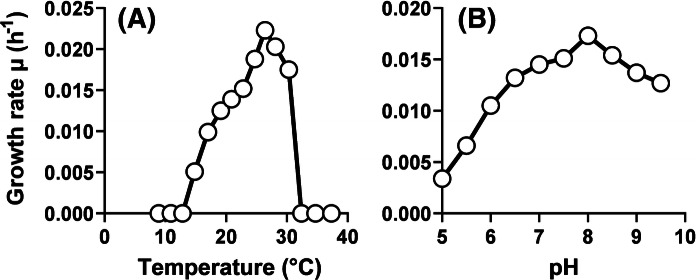
Temperature and pH optimum of strain Pan44^T^. The graphs show the average growth rates obtained from cultivation in M1H NAG ASW medium in biological triplicates. Cultivations at different pH values were conducted at 28 °C and cultivations at different temperatures were performed at pH 8.0

In M1H NAG ASW medium, strain Pan44^T^ was found to grow over a range of 15–30 °C, with optimal growth at 26 °C (Fig. 5a). The temperature profile for growth is comparable to that of *P. piriforme* (range: 10–30 °C, optimum: 20–28 °C) (Table 1). In contrast, large differences were observed for the pH range. *P. piriforme* is slightly acidiphilic with a pH optimum of 6.0–6.5, whereas strain Pan44^T^ showed optimal growth under slightly alkaline conditions (pH 8.0). The strain is able to grow over a range of pH 5–10, while maintaining more than 60% of the maximal growth rate at pH 6.0 and 10.0 (Fig. 5b). The notably broad pH range of strain Pan44^T^ might be an indication of fluctuating pH values in its natural environment. The highest observed growth rate of strain Pan44^T^ in M1H NAG ASW medium was established to be 0.022 h^−1^, corresponding to a doubling time of 32 h.

### Genomic characteristics

The genome of strain Pan44^T^ has a size of 6.76 Mb with a G + C content of 63%. Its genome is 7% larger than the *P. piriforme* P3^T^ genome, which has a slightly lower G + C content (59%). The genome is 1 Mb smaller compared to *M. rarisocia* Mal4^T^, but the G + C content is nearly identical. Comparable numbers were observed for protein-coding genes per Mb and coding densities (Table 1). 43% of the putative protein-encoding genes found in strain Pan44^T^ are of unknown function, which is in the range of 40–55% calculated for most of the planctomycetal genomes sequenced so far (Bordin et al. 2018). With 2814 hypothetical proteins out of a total number 5050 protein-encoding genes (56%) this number is notably higher in *P. piriforme*. The number of tRNAs in the three strains is comparable. Strain Pan44^T^ and *M. rarisocia* Mal4^T^ harbour two copies of the 16S rRNA gene, while a single 16S rRNA gene was found in *P. piriforme* P3^T^.

Although displaying similarities in cell morphology and genome properties, significant differences between the three compared strains were observed, e.g. with regard to colony colour, pH range and optimum, number of hypothetical proteins, the unusual pine cone texture of the cell surface and length of the stalk. Together with the results of the phylogenetic analysis, the data justifies delineation of strain Pan44^T^ from the genera *Maioricimonas* and *Planctomicrobium*. Hence, we conclude that the novel isolate Pan44^T^ (= DSM 29405^T^ = LMG 29788^T^) represents a novel species belonging to a novel genus, for which we propose the name *Caulifigura coniformis* gen. nov., sp. nov.

### *Caulifigura* gen. nov.

*Caulifigura* (Cau.li.fi.gu’ra. L. masc. n. *caulis* a stalk, stem; L. fem. n. *figura* a form, a figure; N.L. fem. n. *Caulifigura* a bacterium shaped like a stalk).

Members of the genus have a Gram-negative cell envelope architecture, are aerobic, mesophilic, neutrophilic to alkaliphilic and heterotrophic. Cells lack pigmentation, divide by budding and produce matrix or fimbriae originating from one of the cell poles. The genus belongs to the family *Planctomycetaceae*, order *Planctomycetales*, class *Planctomycetia*, phylum *Planctomycetes*. The type species of the genus is *Caulifigura coniformis.*

### *Caulifigura coniformis* sp. nov.

*Caulifigura coniformis* (co.ni.for’mis. L. masc. n. *conus* a pine cone; L. masc. adj. suff. -*formis* –like, in the shape of; N.L. fem. adj. *coniformis* shaped like a pine cone, describing the morphology of the cells).

In addition to the genus characteristics, cells are pear-shaped (average size of 1.3 ± 0.2 × 1.0 ± 0.1 µm), occur as single cells, rosettes or larger aggregates and have a characteristic textured cell surface resembling a pine cone. Cells form long stalks. Cells of the type strain grow over ranges of 10–30 °C (optimum 26 °C) and pH 5.0–10.0 (optimum 8.0). Colonies are white. The genome size of the type strain is 6.76 Mb with a G + C content of 63.2%.

The type strain is Pan44^T^ (= DSM 29405^T^ = LMG 29788^T^), isolated from a red biofilm in a hydrothermal are close to the island Panarea, Italy in September 2013. The type strain genome (acc. no. CP036271) and 16S rRNA gene sequence (acc. no. MK554532) are available from GenBank.
